# Donate or Not to Donate—Willingness to Donate and Accept Donor Human Milk

**DOI:** 10.3390/nu17142359

**Published:** 2025-07-18

**Authors:** Yael Lahav, Elad Harison

**Affiliations:** 1 School of Industrial Engineering and Management, Shenkar College of Engineering and Design, Ramat Gan 5290002, Israel; eladha@shenkar.ac.il; 2 Department of Management, Bar Ilan University, Ramat Gan 5290002, Israel

**Keywords:** breast milk donation, donor milk banks, consumer behavior, public health management, behavioral analytics, social support systems, service accessibility, healthcare disparities

## Abstract

**Background/Objectives**: Breast milk donation is increasingly recognized as a vital public health resource, providing optimal nutrition for infants who cannot be breastfed by their biological mothers. Human milk banks play a crucial role in supporting infant health, particularly for preterm and medically vulnerable infants. Despite its recognized benefits, the practice of breast milk donation and utilization remains influenced by a complex interplay of individual, social, and cultural factors. **Methods**: This study systematically examines how demographic and sociocultural variables are associated with attitudes and behaviors related to breast milk donation. Data were collected from 1223 Israeli mothers via questionnaires distributed through WhatsApp groups between April and May 2025. By analyzing the questionnaire results, the research identified key predictors of willingness to donate and use donated milk, as well as patterns of awareness and perceived support within different population groups. **Results**: Higher education (*χ*^2^ = 12.87, *p* = 0.0016) and settlement type (*χ*^2^ = 83.096, *p* = 0.000) were significantly associated with greater willingness to donate breast milk. Income level had no effect on donation behavior, though higher-income participants were more open to its use (χ^2^ = 86.838, *p* = 0.000). Lifestyle also influenced perceptions of social support (*F*(2, 1220) = 259.4036, *p* < 0.001) and cultural support (*F*(2, 1220) = 601.2383, *p* < 0.001) of milk donation and use. Significant correlation was found between perceived cultural and social support (*t* = 5.140, *p* = 0.000), emphasizing their interrelated influence on milk donation attitudes. **Conclusions**: The findings assist in guiding the development of public health policies, as well as targeted awareness campaigns, to promote equitable access to donor milk services across varied communities. From the public health perspective, the findings of this study can orientate campaigns that encourage both donation and use of breast milk focusing on segments of the population in which the level of awareness is relatively low.

## 1. Introduction

Human milk is widely recognized as providing optimal nutrition and significant health benefits for infants [1]. Its components, such as immune factors that include lactoferrin, immunoglobulin A, and human milk oligosaccharides, along with a diverse population of bacteria, contribute to establishing the infant’s gut microbiome, which has lifelong implications for health and immune function [2]. Beyond nutritional advantages, human milk offers protection against respiratory and gastrointestinal tract infections, allergic diseases, and reduces the risk of conditions such as diabetes, obesity, and inflammatory bowel disease [3]. To maximize these benefits, the recommended regimen for exclusive breastfeeding should commence within the first hour of birth and should be continued for six months, later incorporating breastfeeding with complementary feeding up to two years and beyond. However, there are situations where the mother’s own milk is unavailable or insufficient to meet the infant’s needs [4,5]. In such circumstances, international health authorities such as the World Health Organization (WHO) and the American Academy of Pediatrics (AAP) recommend pasteurized donor human milk as the best nutritional alternative, particularly for vulnerable infants, such as those born preterm or with low birth weight [6]. This recommendation is based on evidence showing benefits such as a decreased risk of necrotizing enterocolitis for very low birth weight infants receiving pasteurized donor human milk [7]. Donor human milk (DHM) is thus considered the next best option to ensure that infants receive the benefits of a human milk diet [8].

The growing recognition of DHM’s importance has led to an increasing number of human milk banks (HMBs) being established around the world. This growth is particularly notable in low and middle income countries, aiming to facilitate the collection, processing, and distribution of DHM. To illustrate, in 2020 there were 756 HMBs in 66 countries, a significant increase from approximately 500 in 2016. Europe and the U.S. have the largest number of HMBs, while 70 HMBs were reported to operate in Africa and 44 in Asia at that time [6]. However, despite this expansion of HMBs, a significant challenge remains: the needs of vulnerable infants for DHM often exceed the available supply [9]. This supply constraint highlights critical issues related to the collection process, specifically the willingness of lactating women to donate their milk to HMBs.

The Israeli National HMB was established in 2018 by the national emergency organization Magen David Adom (MDA) and it is regulated by the guidelines for HMBs issued by the Ministry of Health. In addition to the National HMB, human milk can be purchased from private sellers via social networks, although this milk is unregulated. MDA (2024) [10] estimates that the total number of Israeli mothers donating DHM is approximately 1000 women, i.e., less than 1% of the potential donors.

The foundation of the National HMB was motivated by the Remedia case in 2003, in which an Israeli company began offering a new formula that lacked the sufficient quantity of vitamin B1, causing the death of four infants and various long-term damages to more than sixty others [11]. The Remedia case triggered a “food scare” that increased breastfeeding to reduce the reliance on infant formula [12].

Understanding the factors that influence women’s decisions to donate human milk is crucial for the successful operation and sustainability of HMBs and for addressing the global supply shortage. Thereby, the willingness to donate human milk and how it is impacted by various personal and societal attributes are essential as they affect DHM supply to HMBs. The degree of this willingness may be influenced by a complex interplay of factors, including socio-economic attributes [13,14], personal beliefs [6,15], experiences and external support systems [16,17].

Thereby, this paper aims to examine how different social and personal attributes of mothers affect their willingness to donate DHM or to use it. Understanding these effects is essential both for improving DHM donation and use rates and for shaping effective public health policy, strategies and educational campaigns that promote safe and equitable access to DHM in various segments of the population. By identifying facilitators and barriers to milk donation and use, this study assists in enhancing the effectiveness and access of HMBs to broader populations, thereby improving the prospects of infant nutrition and care.

## 2. Literature Review

Our study explores how various attributes influence the willingness of mothers to donate or use DHM. Decisions around milk donation, like those concerning infant feeding, involve multiple issues: consideration or resistance to donation, when and under what circumstances donations take place, and the use of DHM for infants. By assessing the role of key attributes in this process, the paper aims to provide a deeper understanding of the motivations and barriers underlying milk donation behavior and DHM consumption decisions. 

One of the most frequently studied factors in relation to milk donation is the education level of mothers. Education is often associated with health literacy, autonomy in decision-making, and increased access to information—all of which may influence a mother’s decision to donate milk. For instance, in Nigeria, maternal educational level had significantly impacted the willingness to donate or accept DHM. A higher proportion of women with secondary and post-secondary education were not willing to donate human milk in comparison to women possessing other educational levels [5]. However, a study in Uganda found that women with graduate degrees were more likely to donate human milk than non-graduates. This study also found that having a spouse with a graduate degree increased the likelihood of donating milk [6]. These findings reflect differences in educational levels, cultural contexts and awareness levels. Similarly, a study in Vietnam found that having higher education was associated with donating a larger volume of DHM [14]. Its findings suggest that while the impact of education on the initial decision to donate might be complex and context-dependent, it may positively influence the capacity or ability to sustain donation at higher volumes once the decision to donate DHM is positively made. As this body of research presents conflicting findings and conclusions (mostly from low-income countries), the following hypothesis is constructed:

**Hypothesis 1:**  *Willingness to donate human milk increases with the level of education.*

Income is another socio-economic variable that may shape attitudes toward both donation and use of DHM. To a large extent, income may influence a woman’s access to healthcare services, awareness of milk banks, and ability to engage in donation or seek out DHM for her infant. Studies on human milk donations mostly focus on the national level income, namely the behavior of DHM donors mostly within low- and middle-income countries [18]. However, the impact of differences in the personal income of individuals and their willingness to donate milk has largely been overlooked in the literature on DHM and HMBs. Nonetheless, few studies that examined the impact of personal and household income on the willingness to donate milk were carried out. Palmquist and Doehler [19] found that middle-income, college educated mothers in the U.S. were more willing to donate milk. Oreg and Negev [20] reported that no significant personal income effects were found among Israeli milk donors. Ramachandran et al. [3] found no significant household income effects among Malaysian mothers in terms of their willingness to donate milk. Despite the importance of studying these aspects due to the economic and nutritional outcomes that this decision possesses over the use of infant formulas, in recent years no studies were conducted to reveal these relations (with the exception of [19], which was carried out over a decade ago). Further, Fang et al. (2021b) [21] argue that “the motivations behind donating human milk remain under-researched.” Thereby, the following hypotheses were proposed:

**Hypothesis 2. (a):**  *Willingness to donate DHM increases with income.*

**Hypothesis 2. (b):**  *Willingness to use DHM increases with income.*

A limited body of literature was dedicated to the effects of the location of women (in central or remote regions) on their willingness to donate milk. Nonetheless, geographic location was identified as a significant variable in accessing DHM and engaging with milk banks. Specifically, the distinction between central and peripheral regions was found as a key factor in prior studies in shaping awareness, affecting accessibility of DHM and influencing the participation of mothers in milk donation programs. In particular, ref. [8,14] found that Vietnamese mothers from remote provinces are less likely to donate milk in comparison to residents of the central Da Dang province. However, their study attributes these findings to the limited access to medical services in this province and the reliance of HMBs mostly on women that are hospitalized there as potential donors of DHM. Varer Akpinar et al. (2022) [22] studied both local Turkish and refugee women in the remote and rural Aydın region. They concluded that some of the mothers are more motivated to donate DHM and some are more reluctant to do so. Interestingly, in both cases their decisions are motivated by their religious beliefs and their own understanding and interpretation of their religious duties, as well as by their cultural values. Nonetheless, these studies did not compare the willingness to donate milk between women who live in central regions vs. those who live in rural regions, a gap that the following hypothesis addresses:

**Hypothesis 3:**  *Awareness of HMBs is greater among women in central regions when compared with their peers in peripheral regions.*

Beyond centrality, settlement type (i.e., urban vs. rural settlement) may be a key factor affecting milk donation. In general, women residing in urban areas benefit from closer proximity to HMBs, better transportation infrastructure and more robust healthcare systems, in comparison to women residing in rural areas. Consequently, the likelihood of women in urban areas to access donation facilities is greater than their peers in rural areas. Studies on the on DHM identified the distance to HMBs or to designated collection points as a significant barrier to donation [23]. This distance often imposes logistics challenges, particularly transportation to and from the HMB, which can be time-consuming and costly for potential donors. Women residing in peripheral regions have to travel greater distances and confront more substantial logistics challenges to access donation facilities compared to women in more central locations [24]. Proposed strategies to address these barriers, such as opening additional collection points in remote regions or providing transportation or pickup services suggest that geographical disparity translates into donation feasibility, as it impacts access of donors to HMB facilities [25]. Further, the comparison between hospital-based and community-based donors revealed distinct donation patterns: community donors, who might be situated further from central hospital HMBs, donated larger volumes over longer periods but experienced different access challenges. Being a community donor was associated with higher donation volume [26]. 

These findings, highlighting differences between donor groups with potential limited access to DHM and to donation facilities by their location, support the notion that practical, location-dependent factors influence donation. Therefore, based on the documented barriers associated with distance and observed differences in donation patterns potentially linked to geographical access, we hypothesize that:

**Hypothesis 4:**  *Willingness to donate DMH is greater among women from urban regions when compared with their peers in rural regions.*

Awareness of the benefits of DHM and HMBs plays a crucial role in shaping attitudes and behaviors toward both milk donation and use, and may vary due to differences in geographical and social accessibility to information. In different countries, awareness and knowledge of DHM and HMBs are often low among women, with this lack of knowledge considered a major barrier to donation [1]. Women’s concerns about DHM, such as contamination, disease transmission, and cost, are often associated with lack of knowledge and awareness of DHM benefits and processing procedures. Hesitation to use DHM can result from misinformation and lack of education regarding its safety, benefits, and use [2]. Previous studies highlight particularly low awareness in certain regions, such as Southwest Nigeria, where 78% of participants reported never having heard about HMBs [5]. Similarly, a study in Iran found that only 13% of participants had heard about HMBs, noting that mothers had very limited knowledge about their use. These findings were attributed to the relatively recent introduction of HMBs in Iran and the lack of programs promoting milk donation and informing mothers about HMBs [17]. Similar lack of awareness was reported in Çeştepe, Aydın, a rural region in Turkey, in which 75% of the women reported they had never heard of HMBs. These studies emphasize the importance of facilitating knowledge and positive attitudes among women in rural regions towards the use of HMBs and donating DHM. Women in rural Turkey who heard about HMBs (25.1%), received this information from social media (65.7%), healthcare professionals (20.9%), and friends (13.4%). However, none of the women in the rural Turkish regions studied had specific knowledge about how HMBs function [22]. A hospital-based study in Uganda found that more than 50% of respondents were aware of HMBs, and over 75% of mothers at the studied hospital were willing to donate, a finding which correlates with positive perception, awareness and exposure to DHM activities at the hospital [27].

The documented low levels of awareness in various settings, particularly the very low awareness reported in the rural Turkish regions, and the challenges in reaching rural populations, support the notion that awareness of HMBs likely differs between women living in urban vs. rural locations. This location-based can be attributed to the absence of HMB systems, limited access to information channels, and greater geographical distance to facilities. Therefore, the following hypothesis is proposed:

**Hypothesis 5:**  *Awareness of HMBs is greater among women from urban regions when compared with their peers in rural regions.*

Studies from diverse regions worldwide demonstrate how cultural and religious beliefs act as significant barriers to both the donation and acceptance of human milk [28]. A prominent example is the concept of “milk kinship” in Islam, where the sharing of milk can create familial bonds that prohibit marriage between individuals, leading to hesitation among Muslim mothers in countries such as Malaysia, Turkey, and Iran regarding donation or acceptance of DHM from anonymous donors [29]. Beyond the formal religious doctrine, broader cultural beliefs such as societal taboos, distrust of unknown individuals, fears of transmitting undesirable traits or diseases, and concerns about hygiene also deter donation and acceptance of DHM. For instance, some women express fears that donating milk could cause harm or transmit traits like criminality. Conversely, religious beliefs can also serve as facilitators, where donating milk is perceived as a compassionate “good deed” to help a child in need. The varied and context-specific influence of these moral and cultural factors on attitudes towards milk donation is evident across diverse populations. Therefore, we hypothesize that:

**Hypothesis 6:**  *Cultural and religious background affects moral and cultural attitudes of women toward milk donation.*

The cultural and religious views mentioned above are not restricted only to individuals, but are often shared within families and communities [15,30]. Consequently, these shared beliefs can manifest as family stigma, negative rumors, lack of support from family members, or general societal taboos and lack of acceptance, directly impacting the social environment surrounding a potential donor or recipient [31,32,33,34,35]. These insights suggest that a woman’s perception of the acceptability of milk donation within her social circles is influenced by her cultural and religious context. Therefore, the following hypothesis is presented:

**Hypothesis 7:**  *Cultural and religious background affects the perceived social support for milk donation.*

Social support from family, particularly spouses or partners, friends, and the wider community, can act as significant facilitators for milk donation [36,37]. Encouragement and information from HMBs are also crucial cultural enablers, influencing mothers’ willingness and confidence to donate. Hence, by understanding how mothers perceive the support and acceptance of milk donation within their social, religious and cultural spheres, effective strategies that increase donation rates and ensure equitable access to DHM can be developed and introduced to the public [38]. Thereupon, the following hypothesis is presented:

**Hypothesis 8:**  *Correlation between perceived cultural support and social support for milk donation exists.*

## 3. Materials and Methods

This study examines how demographic and sociocultural variables affect attitudes and behaviors related to breast milk donation in a diverse population of 1223 participants. Data was collected via questionnaires distributed through WhatsApp groups between April and May 2025 to Israeli mothers of 22–45 years old who have at least one child. Following the methodology described in [39] that conducted a questionnaire-based study in Israel, the sample size was based on a small effect size of 0.02, *α*  =  0.05 and a power of 80%. Hence, the calculated sample size is 660, while the actual number of respondents was 1223.

Statistical analyses were conducted using R (version 4.4.2). The dataset included a range of demographic, socio-economic and cultural variables, as well as attributes affecting human milk donation behaviors and attitudes. Categorical variables, such as education level, income, settlement type, geographic region, and lifestyle (i.e., cultural and religious background in terms of secular, traditional and religious beliefs) were collected on a Lickert scale ranging from 1 to 5 to measure the perceived cultural support and social support for breast milk donation. Key variables, such as education, income and region, were used to examine disparities in willingness to donate and use DHM. Settlement types were considered critical for understanding geographic and contextual influences on awareness of HMBs. Cultural dimensions are explored through variables such as cultural and religious background, lifestyle, and moral and cultural attitudes toward milk donation. These factors provide insights into the broader social context of milk donation and women’s decision-making.

### Statistical Analysis

Data preprocessing included converting categorical variables into factors and conducting preliminary descriptive analyses. Chi-squared tests were used to examine associations between the following variable pairs: education and prior breast milk donation, income and usage of donated milk, region and awareness of milk banks, settlement types and donation behavior and settlement types and awareness of milk banks.

One-way ANOVA was used to examine the impact of lifestyle on both perceived cultural support for breast milk donation and perceived social support for donation.

Pearson correlation was used to examine the relationship between perceived social support and perceived cultural support. All statistical analyses were performed using significance level of *p* < 0.05.

## 4. Results

This study examined eight hypotheses related to socio-demographic, cultural, and psychological factors influencing attitudes and behaviors surrounding milk donation and use. The sample included 1223 participants of 22–45 years old. The age distribution included 406 respondents in their twenties (33.2%), 512 respondents in their thirties (41.8%) and 305 respondents in their forties (25%), with a mean age of 33.6 years (SD = 6.5). The marital status distribution included 742 married (60.6%), 146 divorced (12%), 52 widowed (4.3%) and 283 single mothers (23.1%). Income of participants included the following distribution: 205 respondents (16.8%) earned under 8000 New Israeli Shekels, i.e., NIS (2200 US Dollars, i.e., USD), 320 respondents (26.2%) earned between 8001 and 15,000 NIS (2201–4200 USD), 421 respondents (34.4%) earned between 15,001 and 20,000 NIS (4201–5600 USD) and 277 respondents (22.6%) earned above 20,001 NIS (5601 USD) (see Table A2a). The education level was distributed as follows: 189 respondents (15.5%) completed certificate studies, 639 respondents (52.2%) had a Bachelor’s degree and 395 respondents (32.3%) held a Master’s or a Doctoral degree (see Table A1a).

The regional distribution included 390 respondents (31.9%) from the Center, 384 respondents (31.4%) from the North and 449 respondents (36.7%) from the South of the country (see Table A3a). The geographical distribution included 620 respondents (50.7%) that reside in urban regions, 385 respondents (31.5%) living in towns and 218 respondents (17.8%) residing in rural regions (see Table A4a). Lifestyle distribution included 609 respondents (49.8%) identifying themselves as secular, 372 respondents (30.4%) as traditional and 242 respondents (19.8%) as religious (see Table A5a). See Table 1 for sociodemographic characteristics of the respondents.

A Chi-square test revealed a significant relation between education level and willingness to donate breast milk (*χ*^2^ = 12.87, *p* = 0.0016) (see Table A1b). Therefore, participants with higher education levels (Master and PhD) were more likely to express willingness to donate compared to certificate holders (see Table A1c).

Two non-parametric Kruskal–Wallis tests were conducted to explore whether income level influences women’s willingness to donate or receive donated human milk. Income was defined as an ordered categorical variable with four levels: Under 8000 NIS, 8001–15,000 NIS, 15,001–20,000 NIS, and above 20,001 NIS.

A Kruskal–Wallis rank sum test was applied to assess differences in self-reported donation behavior across income groups. The test result was not found as statistically significant (χ^2^ = 2.991, *p* = 0.3931). This result suggests that there is no significant relation between income level and willingness to donate human milk among participants in this sample (see Table A2b).

A second Kruskal–Wallis test examined willingness to use DHM by income level. This test revealed a statistically significant difference across groups (χ^2^= 86.838, *p* = 0.000). The result indicates that income is significantly associated with individuals’ willingness to use DHM (see Table A2c). Following these findings, post hoc pairwise comparisons were conducted using the Wilcoxon rank-sum test with Holm adjustment. The results revealed several statistically significant differences, by which participants in the 15,001–20,000 NIS group were significantly more likely to have used DHM than those in the Under 8000 NIS (*p* < 0.001) and 8001–15,000 (*p* < 0.001) groups. Similarly, participants in the Above 20,001 NIS group were significantly more likely to use DHM than those in the Under 8000 (*p* < 0.001) and 8001–15,000 (*p* < 0.001) groups. No statistically significant difference was found between the 15,001–20,000 and Above 20,001 NIS groups (*p* = 0.72), nor between the Under 8000 and 8001–15,000 groups (*p* = 0.32). These findings confirm that higher-income participants (15,001 NIS and above) have greater willingness to use DHM, particularly in comparison to lower-income groups (see Table A2d,e). The statistical analysis presents no significant variation in awareness of HMBs was found across regions (*χ*^2^ = 2.98, *p* = 0.225), indicating similar awareness levels among participants from different regions (see Table A3b). Additionally, the variable of settlement type was found to significantly affect willingness to donate breast milk (*χ*^2^ = 83.096, *p* = 0.000) (see Table A4b). Urban residents were more likely to express willingness to donate compared to those residing in towns or rural regions (see Table A4c). However, no significant relation was observed between settlement type and awareness of HMBs (*χ*^2^ = 0.58, *p* = 0.75) (see Table A4d).

One-way ANOVA revealed a highly significant effect of lifestyle on attitudes of women toward milk donation (*F*(2, 1220) = 601.2383, *p* < 0.001) (see Table A5b). Secular and traditional participants reported higher propensity to donate in comparison to religious participants (Table A5c). A significant effect of lifestyle on perceived social support was observed with secular participants reporting the highest levels of social support, followed by traditional and religious participants (*F*(2, 1220) = 259.4, *p* < 0.001) (see Table A5d,e).

Finally, Pearson’s correlation analysis indicated a weak but significant positive correlation between perceived cultural support and perceived social support for breast milk donation (*r* = 0.15, 95% *CI* [0.09, 0.20], *p* < 0.001) (see Table A6). This result indicates that individuals who possess stronger cultural support for milk donation also have higher levels of social support and vice versa. The hypotheses and the results of the study are summarized in Table 2.

## 5. Discussion

Our analysis sheds light on important insights relates to the motivations and barriers that shape behaviors surrounding milk donation and use of DHM. The study examines the influence of socio-demographic, cultural, and psychological factors on mothers’ willingness to donate or use DHM.

The results of Hypothesis 1 indicate that education plays a significant role in influencing milk donation behavior. Participants with higher education levels (Master and PhD) were more likely to express willingness to donate than certificate holders. This finding supports earlier studies carried out in Uganda and Vietnam [6,14] in which education was found to foster greater awareness and trust in provision, processing and use of DHM via HMBs. However, this finding is opposite to the conclusions of a study conducted in Nigeria [5] that found that higher education is associated with greater reluctance to donate DHM. These contradictory findings suggest that while education can empower women with knowledge, its influence is heavily mediated by cultural, contextual and religious factors. These insights highlight the need for culturally tailored educational campaigns that not only inform women about DHM but do so in a way that aligns with their social and moral frameworks.

Our study did not find any statistically significant relations between income level and willingness to donate DHM, suggesting that altruistic motivations for donation are independent of the economic status of mothers, therefore negating Hypothesis 2a. This result is consistent with similar findings from a study conducted in Malaysia [3], which did not find any relations between household income and willingness to donate. However, the level of income was found to have significant impact on willingness to use DHM, thereby confirming Hypothesis 2b. Higher-income participants expressed higher degrees of willingness to use DHM, especially those earning above 15,000 NIS per month (above 4201 USD). This finding indicates that lower-income groups may face barriers to accessing and using DHM due to real or perceived costs, limited healthcare access or lack of information, although the decision to donate may is not influenced by economic factors.

Geographical factors play a significant role in shaping behaviors and attitudes toward donation and use of DHM. However, our study found that awareness of HMBs did not significantly differ across regions, thereby negating Hypothesis 3. This finding contradicts the conclusions of [7,8,22], by which central regions differ in willingness to donate DHM in comparison to peripheral regions. It is possible to explain the contradiction between the findings of this study and prior studies by the difference in country size of Israel vs. Vietnam and Turkey. Additionally, the economic differences between these countries affect access to qualitative medical services to the extent that they are available throughout Israel, while former studies indicate that the access to these services are limited in the peripheral regions of Vietnam and Turkey.

Settlement type had significant impact on willingness to donate. This study found that women living in urban regions were more willing to donate DHM than those residing in towns or rural regions, thereby confirming Hypothesis 4. This result highlights the persistent logistics and infrastructural challenges that women in less central regions confront, including transportation issues, longer travel distances to collection sites or limited contact with hospital-based HMBs. Notably, no significant differences in the level of awareness of HMBs were found between women from urban and rural regions, thereby negating Hypothesis 5. This finding implies that information about HMBs is relatively well communicated on a national level, which can be attributed to effective communication by public health authorities mostly via medical professionals and digital platforms.

Cultural and religious backgrounds were found to significantly influence attitudes toward DHM, thereby confirming Hypothesis 6. Additionally, this study found that the groups of secular and traditional participants reported greater cultural and social support for milk donation than religious ones, thereby confirming Hypothesis 7. This finding is particularly interesting as it reflects the impact of religious background. Depending on context, cultural and religious beliefs may either hinder or encourage donation, viewed as a moral duty by some, but raising concerns about identity or purity for others. Despite the fact that all participants were Jewish women and the Islamic concept of milk kinship [29] does not exist in Jewish scholarship, these women had attitudes toward donation and use of DHM that were similar to their Muslim peers. These findings highlight the need for culturally sensitive messaging and the involvement of religious and community leaders to promote DHM in line with shared values.

A significant positive correlation, though weak, between perceived cultural and social support for milk donation was observed, affirming Hypothesis 8. The identified correlation suggests that cultural acceptance can reinforce interpersonal and familial support, and vice versa. This result highlights the association between social and cultural factors affecting maternal health behaviors and choices. Therefore, strategies that aim to increase the donation and use of DHM should address both individual beliefs and the wider social environment and contexts.

In summary, the findings of this research suggest that willingness to donate or use DHM is not determined by a single factor, but rather by a multifaceted interplay of educational, economic, geographic, cultural, and social influences. Consequently, efforts to improve the accessibility and availability of DHM should implement multidimensional strategies that address various segments in the population. Public health initiatives should include educational campaigns tailored to different religious and cultural groups, facility improvements that reduce geographical disparities and financial support mechanisms for families that cannot afford the use of DHM.

## 6. Conclusions

This study highlights key socio-demographic and cultural attributes affecting breast milk donation behavior, offering important insights for public health policy and donor milk program development.

The findings indicate that higher education and income levels, as well as settlement type, positively affect the willingness to donate and use DHM. These results align with the conclusions of [11] that the educational level of mothers affects the choices of feeding their infants. Additionally, higher income individuals were more likely to use DHM, indicating the existence of income-based disparities in accessing healthcare settings and informational resources. These findings emphasize the importance of the initiation of pro-active operations, such as targeted campaigns, by public health authorities to ensure equitable access and participation across different socio-economic groups.

Settlement types were also identified as a significant predictor, with urban residents more likely to donate breast milk in comparison to residents of rural regions. However, no significant differences in awareness of HMBs were found between residents of central versus peripheral regions or settlement types, suggesting that the geographic location does not affect the degree of awareness. Nonetheless, other factors, such as healthcare system integration or exposure to public health messages, may play a more substantial role in shaping public awareness.

Beyond the socio-demographic factors, cultural and religious identity strongly influences attitudes toward milk donation. Participants who identified themselves as secular, traditional or religious were found to have significant differences in the reported level of cultural and social support for donating breast milk. In particular, the results indicate that cultural and moral values may shape donation behavior and acceptance. Following these insights, culturally sensitive public health messaging and community engagement strategies are necessary to increase awareness and acceptance of milk donation across diverse population groups.

Although this study relies on questionnaires and statistical analysis, the cultural, societal, and religious aspects of DHM donation and use call for further qualitative research. Such methods can offer deeper insights into the motivations, concerns, and experiences of donors and recipients. A complementary qualitative research direction may include interviews with policy makers and professionals to support identifying potential obstacles in broader DHM donation and use among various population segments. Additional venues for further research should look into the difference between the effect of income on the willingness to use DHM vs. the finding that income has no significant effect on the willingness to donate milk. The similarities in awareness of HMBs across regions in Israel may be explained either by the relatively small size of the country (in comparison to Turkey and Malaysia that were reviewed in this paper) or due to the centralized policy of operating a National HMB. Therefore, further research, backed by a policy study of the Israeli DHM guidelines is required.

Some of the potential limitations of this study include the distribution of questionnaire via WhatsApp, which may result in a selection bias due to lack of ownership of smartphones in small segments of the Israeli society, such as ultra-Orthodox women, immigrants and lower-income individuals, which could affect to some extent the representativeness of the sample. Additionally, this study has mostly evaluated the impact and the results of behavioral attributes of mothers donating and using DHM. However, further research can assess the effects of personal attributes, such as age, marital status, number of children and status of employment on mothers’ decisions.

Additionally, implementation of qualitative methods may shed light on the complex social and emotional dynamics involved in the donation and use of DHM. Such extended research designs can benefit our understanding of how attitudes and behaviors related to milk donation change over time, particularly in response to targeted awareness campaigns or institutional programs. Finally, future studies should examine the influence of the recommendations by healthcare providers on the decisions of individuals to donate or utilize DHM, as professional guidance may play a major role in shaping public perceptions and behaviors.

## Figures and Tables

**Table 1:** Sociodemographic characteristics of the respondents.

Variable	Description	Distribution	%
Age	Mother’s age	22–30 31–40 41–45	33.2% 41.8% 25%
Marital status	Status of the respondent	Married Divorced Widowed Single	60.6% 12% 4.3% 23.1%
Income	Monthly income in NIS	Under 8000 Between 8001–15,000 Between 15001–20,000 Above 20,001	16.8% 26.2% 34.4% 22.6%
Education	Educational level	certificate studies Bachelor’s degree Master or PhD	15.5% 52.2% 32.3%
Regional	Geographical location	Center North South	31.9% 31.4% 36.7%
Settlement type	The type of settlement in which the respondent lives	Urban Town Rural	50.7% 31.5% 17.8%
Lifestyle	Cultural and religious background	Secular Traditional Religious	49.8% 30.4% 19.8%

**Table 2:** Summary of hypotheses and levels of support.

Hypothesis	Description	Result	Level of Support	Justification
H1	Willingness to donate human milk increases with the level of education.	*χ*^2^ = 12.87, * p* = 0.0016	Strongly supported	Significant positive relationship; higher education levels linked to greater willingness to donate.
H2a	Willingness to donate DHM increases with income.	*χ*^2^ = 2.991, *p* = 0.3931	Unsupported	No statistically significant relationship found.
H2b	Willingness to use DHM increases with income.	*χ*^2^ = 86.838, *p* = 0.000	Strongly supported	Significant differences; higher-income groups more willing to use DHM.
H3	Awareness of HMBs is greater among women in central regions when compared with their peers in peripheral regions.	*χ*^2^ = 2.98, *p* = 0.225	Unsupported	No significant regional differences in awareness.
H4	Willingness to donate DMH is greater among women from urban regions when compared with their peers in rural regions.	χ^2^ = 83.096, *p* = 0.000	Strongly supported	Significant differences; urban residents more willing to donate.
H5	Awareness of HMBs is greater among women from urban regions when compared with their peers in rural regions.	*χ*^2^ = 0.575, *p* = 0.75	Unsupported	No significant differences were found between women from urban and rural regions.
H6	Cultural and religious background affects moral and cultural attitudes of women toward milk donation.	*F*(2, 1220) = 601.24, * p* < 0.001	Strongly supported	Secular and traditional participants reported higher propensity to donate.
H7	Cultural and religious background affects perceived social support for milk donation.	*F*(2, 1220) = 259.4, * p* < 0.001	Strongly supported	Significant differences in social support by religious and cultural background. Secular participants had the highest social support, followed by traditional and religious participants.
H8	Correlation exists between perceived cultural support and social support for milk donation.	*r* = 0.15, * p* < 0.001	Moderately supported	Significant but weak positive correlation between cultural and social support.

## Data Availability

Data is available from the authors upon request.

## Abbreviations

The following abbreviations are used in this manuscript:

AAP	American Academy of Pediatrics
DHM	Donor Human Milk
HMB	Human Milk Banks
MDA	Magen David Adom
NIS	New Israeli Shekels
USD	US Dollars
WHO	World Health Organization

## Appendix A

**Table A1:** (**a**) Education frequency distribution; (**b**) Chi-square test—education and willingness to donate; (**c**) proportion willing to donate by education level.

(**a**)	
**Var1**	**Freq**
Certificate studies	189
Bachelor	639
Master/PhD	395
(**b**)	
**Statistic**	**Value**
Chi-squared	12.869
df	2.000
*p*-value	0.0016
(**c**)	
**Education_Level**	**Proportion_Willing_to_Donate**
Certificate studies	0.402
Bachelor	0.521
Master/PhD	0.559

**Table A2:** (**a**) Income frequency distribution; (**b**) Kruskal–Wallis test—willingness to donate milk by income; (**c**) Kruskal–Wallis test—willingness to use of donated milk by income; (**d**) proportion using donated milk by income level; (**e**) post hoc pairwise Wilcoxon tests (Holm-adjusted *p*-values).

(**a**)				
**Var1**	**Freq**			
Under_8000	205			
8001_15,000	320			
15,001_20,000	421			
Above_20,001	277			
(**b**)				
**Statistic**	**Chi-Squared**	**df**		***p*** **-Value**
Kruskal–Wallis	2.991	3		0.3931
(**c**)				
**Statistic**	**Chi-Squared**	**df**		***p*** **-Value**
Kruskal–Wallis	86.838	3		0.000
(**d**)				
**Income_Level**	**Proportion_Used_Donated_Milk**			
Under_8000	0.107			
8001_15,000	0.072			
15,001_20,000	0.309			
Above_20,001	0.296			
(**e**)				
**Group 1**	**Group 2**		**Adjusted *p*-Value**	
8001_15,000	Under_8000		0.31516	
15,001_20,000	Under_8000		0.00000	
Above_20,001	Under_8000		0.00000	
15,001_20,000	8001_15,000		0.00000	
Above_20,001	8001_15,000		0.00000	
Above_20,001	15,001_20,000		0.72023	

**Table A3:** (**a**) Region frequency distribution; (**b**) Chi-square test—region and awareness of milk bank.

(**a**)	
**Var1**	**Freq**
Center	390
North	384
South	449
(**b**)	
**Statistic**	**Value**
Chi-squared	2.983
df	2.000
*p*-value	0.225

**Table A4:** (**a**) Settlement type frequency distribution; (**b**) Chi-square test—settlement type and willingness to donate; (**c**) proportion willing to donate by settlement type; (**d**) Chi-square test—settlement type and awareness of HMBs.

(**a**)	
**Var1**	**Freq**
Urban	620
Town	385
Rural	218
(**b**)	
**Statistic**	**Value**
Chi-squared	83.096
df	2.000
*p*-value	0.000
(**c**)	
**Settlement_Type**	**Proportion_Willing_to_Donate**
Urban	0.644
Town	0.387
Rural	0.376
(**d**)	
**Statistic**	**Value**
Chi-squared	0.575
df	2.000
*p*-value	0.750

**Table A5:** (**a**) Lifestyle frequency distribution; (**b**) ANOVA—cultural attitudes by lifestyle; (**c**) mean cultural attitudes by lifestyle; (**d**) ANOVA—social support by lifestyle; (**e**) mean social support by lifestyle.

(**a**)					
**Var1**		**Freq**			
Secular		609			
Traditional		372			
Religious		242			
(**b**)					
	**Df**	**Sum Sq**	**Mean Sq**	**F value**	**Pr(>F)**
Lifestyle	2	799.849	399.924522	601.2383	1.910463 × 10^−182^
Residuals	1220	811.505	0.665168		
(**c**)					
**Lifestyle**		**Culture Attitudes**			
Secular		4.01			
Traditional		4.08			
Religious		2.01			
(**d**)					
	**Df**	**Sum Sq**	**Mean Sq**	**F value**	**Pr(>F)**
Lifestyle	2	339.1625	169.5812606	259.4036	1.337007 × 10^−94^
Residuals	1220	797.5570	0.6537353		
(**e**)					
**Lifestyle**		**Social_Support**			
Secular		4.06			
Traditional		2.98			
Religious		3.04			

**Table A6:** Pearson correlation—cultural and social support.

**Statistic**	**Value**
Correlation Coefficient (r)	0.15
t	5.14
df	1221.00
*p*-value	0.00
95% CI Lower	0.09
95% CI Upper	0.20
